# Supplementary material for: Optimal Treatment with Cannabis Extracts Formulations Is Gained via Knowledge of Their Terpene Content and via Enrichment with Specifically Selected Monoterpenes and Monoterpenoids
Source: Molecules. 2022 Oct 15;27(20):6920. doi: 10.3390/molecules27206920 (PMC9608144; doi:10.3390/molecules27206920)
Supplement: Supplementary file 1 [file molecules-27-06920-s001.zip › molecules-1945670-supplementary.pdf]

SUPPLEMENTARY ANALYTICAL INFORMATION:

High Performance Liquid Chromatography (HPLC):

Figure S1: HPLC chromatogram – a mixture of cannabinoid standards

Chromatogram and Results

Injection Details

Injection Name:	**SST validation**	HPLC_03	*Run Time (min):*	**15.00**
Vial Number:	**RA5**		*Injection Volume:*	**10.00**
Injection Type:	**Unknown**		*Channel:*	**UV_VIS_1**
Calibration Level:			*Wavelength:*	**220**
Instrument Method:	**HPLC 3 canabinoids**		*Bandwidth:*	**n.a.**
Processing Method:	**Aminolab**		*Dilution Factor:*	**1**
Injection Date/Time:	**18.03.21 12:29**		*Sample Weight:*	**1.00**

Chromatogram

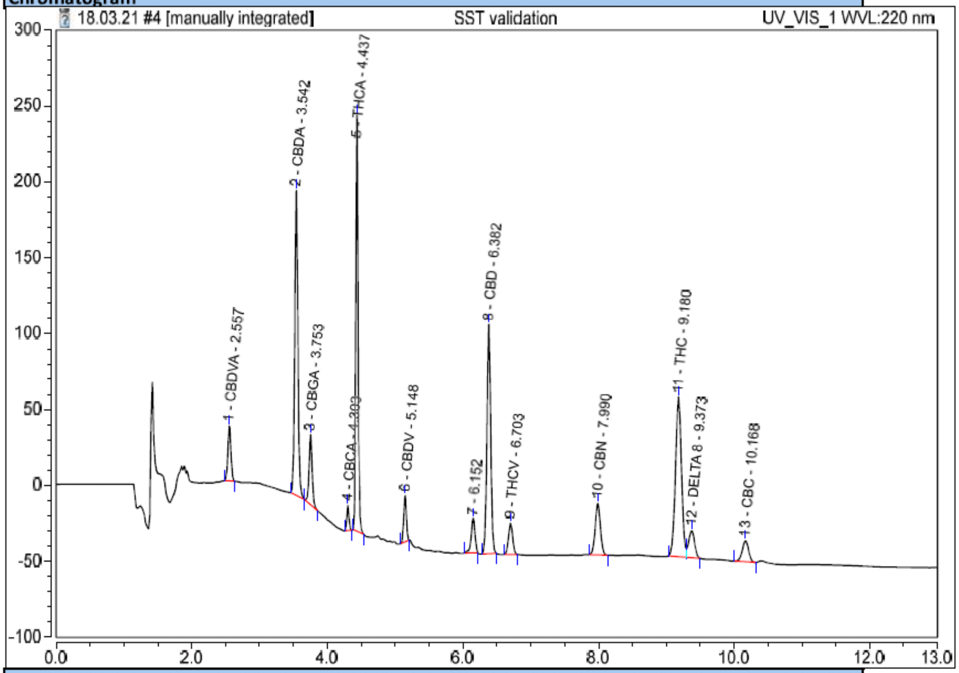

Integration Results

No.	Peak Name	Retention Time min	Area mAU*min	Height mAU	Amount mg/ml	Plates (EP)	symmetry (E	Resolution (EP)
1	CBDVA	2.557	1.8	36.0	0.00	17368	0.99	12.32
2	CBDA	3.542	10.7	200.9	0.01	29346	1.08	2.70
3	CBGA	3.753	2.3	46.1	0.00	40387	1.01	8.36
4	CBCA	4.303	0.6	16.7	0.00	90494	1.07	2.31
5	THCA	4.437	10.4	274.1	0.01	92205	1.09	10.79
6	CBDV	5.148	1.4	30.5	0.00	77699	0.96	11.82
8	CBD	6.382	9.9	151.0	0.02	61297	1.01	3.00
9	THCV	6.703	1.4	20.6	0.00	57549	1.00	10.25
10	CBN	7.990	3.0	34.0	0.00	52222	1.00	7.88
11	THC	9.180	10.9	105.3	0.02	50810	n.a.	1.18
12	DELTA 8	9.373	1.8	17.9	0.00	51621	n.a.	4.79
13	CBC	10.168	1.5	13.9	0.00	58789	0.94	n.a.

Gas Chromatography (GC):

Figure S2: GC chromatogram – Terpene standards

Chromatogram and Results

Injection Details

Injection Name:	**23 Terpenes New SST**	*Run Time (m*	**25.25**
Vial Number:	**94**	*Injection Volu*	**1.00**
Injection Type:	**Unknown**	*Channel:*	**GC_1**
Calibration Level:		*Analyst*	**Talal**
Instrument Method:	**LAB-012-M03-25min**		
Processing Method:	**LAB-012-M03**		
Injection Date/Time:	**17.04.22 21:33**		

Chromatogram

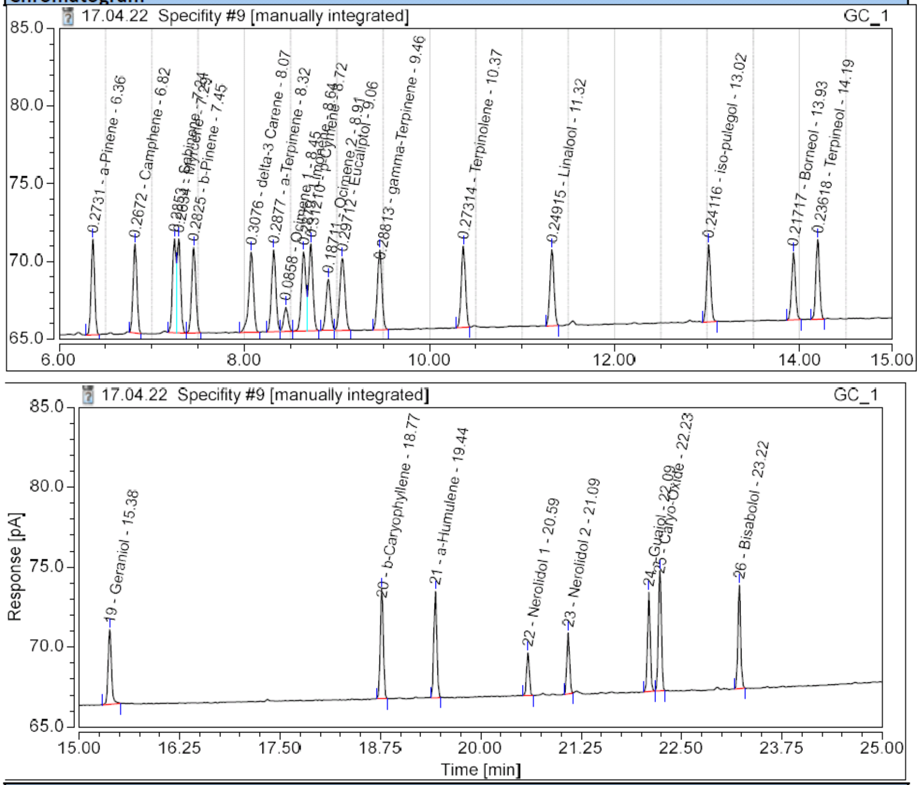

Integration Results

No.	Name	Retention Time min	Area pA*min	Height pA	Amount mg/mL	Plates (EP)	symmetry (E	Resolution (EP
1	a-Pinene	6.36	27261	6.2	n.a.	132332	1.0	n.a.
2	Camphene	6.82	26732	5.7	n.a.	132917	1.0	6.3
3	Sabinene	7.24	28457	6.0	n.a.	n.a.	n.a.	n.a.
4	Myrcene	7.29	26286	6.1	n.a.	n.a.	n.a.	n.a.
5	b-Pinene	7.45	28215	5.5	n.a.	134937	1.0	n.a.
6	delta-3 Carene	8.07	30689	5.2	n.a.	135784	0.8	7.4
7	a-Terpinene	8.32	28738	5.3	n.a.	150012	1.0	2.8
9	Limonene	8.64	28706	5.1	n.a.	129989	n.a.	2.1
10	p-Cymene	8.72	31154	5.6	n.a.	140743	n.a.	0.8
12	Eucaliptol	9.06	29748	4.7	n.a.	127502	1.0	1.6
13	gamma-Terpinene	9.46	28760	5.2	n.a.	181014	1.0	4.2
14	Terpinolene	10.37	27274	5.2	n.a.	248592	1.0	10.5
15	Linalool	11.32	24926	4.9	n.a.	316189	1.0	11.7
16	iso-pulegol	13.02	24102	5.0	n.a.	466487	1.1	21.6
17	Borneol	13.93	21704	4.3	n.a.	488232	1.0	11.8
18	Terpineol	14.19	23613	5.1	n.a.	606906	1.0	3.4
19	Geraniol	15.38	21998	4.7	n.a.	761209	1.1	16.6
20	b-Caryophyllene	18.77	30232	6.9	n.a.	1143292	1.0	48.3
21	a-Humulene	19.44	29492	6.7	n.a.	1222751	1.0	9.5
24	Guaiol	22.09	25493	6.2	n.a.	1832089	1.0	15.8
25	Caryo-Oxide	22.23	31269	7.5	n.a.	1823531	1.0	2.1
26	Bisabolol	23.22	25519	6.5	n.a.	2239251	1.0	15.5

Terpene Standards:

1.1 RESTEK Cannabis Terpenes Standard #1, Catalog No.34095 lot.N0#.: A0172649, Exp. date: May 31, 2023 and lot #A0172251 Exp. date: May 31, 2023 , 2500 μg/mL, Isopropanol, 1mL/ampule.

1.2 RESTEK Cannabis Terpenes Standard #2 Eucalyptol, Caryofyllene Oxide - (2 components) Catalog No.34095 lot.N0#.: A0172649

1.3 MERCK Supelco (+)-Borneol standard solution 2000 μg/mL, Cat. #CRM40901, Lot#LRAD0679, Exp. date:31/10/2024

1.4 RESTEK Alpha Terpineol standard solution 2000 μg/mL, Cat. #33912, Lot #A0180139, Exp. date. 09/2024

1.5 Sabinene, Phyto Lab cat#82343

SUPPLEMENTARY STATISTICAL INFORMATION:

Statistical analysis

An independent samples t-test analysis was conducted with a significance level of 0.05.

Statistical analyses were performed with SPSS 20.0 software (IBM Corp., Armonk, N.Y.).

Terpene content in commercial medical cannabis inflorescences and olive-diluted cannabis extracts (Data presented in Figure 3). An independent samples t-test was performed on three variables: total MONO terpenes, total SESQUI terpenes and total terpenes with one between-subjects variable – type of product (cannabis inflorescences and cannabis diluted extracts).

Terpene content in inflorescences and decarboxylated extracts produced from them (Data presented in Figure 4). An independent samples t-test was performed on 30 variables: 27 individual terpenes, total MONO terpenes, total SESQUI terpenes and total terpenes, with one between-subjects variable – type of product (cannabis inflorescences and cannabis decarboxylated extract). For the purpose of statistical analysis, cases having terpene content of below quantification limit (<200 ppm) were treated as having terpene content of 190 ppm; Terpenes with RT shorten than the RT of β-caryophyllene were regarded within the Total MONO (monoterpenes and monoterpenoids) group, terpenes with RT similar to or longer than that of β-caryophyllene were regarded within the Total SESQUI (sesquiterpenes and sesquiterpenoids) group.

Table S1: Comparisons between individual terpene content in ten inflorescences and decarboxylated extracts produced from them.

	Inflorescences		Decarboxylated extracts					
	M	SD	M	SD	df	t	p	Cohen's d
α-Pinene	1.07	1.61	0.05	0.29	9.01	2.01	p>0.05	0.90
Camphene	0.13	0.28	0.05	0.45	18.00	4.83	p<0.001	2.16
Sabinene	0.13	0.28	0.05	0.45	18.00	4.83	p<0.001	2.16
β-Pinene	0.38	0.34	0.11	0.19	18.00	2.17	p<0.05	0.27
Myrcene	1.51	1.20	0.20	0.52	12.23	3.18	p<0.01	0.97
Carene	0.13	0.28	0.05	0.45	18.00	4.83	p<0.001	2.16
Ocimene	0.13	0.33	0.05	0.45	18.00	4.37	p<0.001	1.96
Limonene	0.78	0.05	0.10	0.15	9.66	2.78	p<0.05	1.24
Terpinolene	0.13	0.28	0.05	0.05	18.00	4.69	p<0.001	2.10
Linalool	0.24	0.16	0.27	0.14	18.00	-0.46	p>0.05	-0.20
RT 13.0*	0.23	0.11	0.18	0.14	18.00	0.79	p>0.05	0.36
RT 14.7*	0.15	0.06	0.11	0.08	18.00	1.41	p>0.05	0.63
Terpineol	0.17	0.09	0.10	0.08	18.00	1.82	p>0.05	0.81
Geraniol	0.14	0.28	0.05	0.45	18.00	4.86	p<0.001	2.16
RT19.1*	0.15	0.05	0.07	0.05	18.00	3.28	p<0.01	1.47
β-Caryophyllene	1.72	1.02	1.34	0.45	12.32	1.09	p>0.05	0.49
α -Humulene	0.48	0.27	0.39	0.13	12.92	0.96	p>0.05	0.43
RT 20.2*	0.26	0.40	0.25	0.35	18.00	0.04	p>0.05	0.02
RT 20.4*	0.20	0.11	0.14	0.07	18.00	1.33	p>0.05	0.60
RT 20.7*	0.14	0.03	0.12	0.04	18.00	1.22	p>0.05	0.55
RT 20.8*	0.16	0.10	0.09	0.05	18.00	1.87	p>0.05	0.23
RT 20.9*	0.29	0.16	0.29	0.20	18.00	-0.12	p>0.05	0.83
RT 21.0*	0.39	0.41	0.33	0.32	18.00	0.37	p>0.05	0.06
Nerolidol	0.16	0.06	0.09	0.06	18.00	2.15	p>0.05	0.17
Guaiol	0.25	0.17	0.02	0.21	18.00	0.31	p>0.05	0.14
Eudesmol*	0.26	0.18	0.24	0.23	18.00	0.23	p>0.05	0.10
Bisabolol	0.86	0.46	0.79	0.56	18.00	0.28	p>0.05	0.13

Data for *Total Terpenes*, *Total MONO* and *Total SESQUI* is presented in the main text.
